# Comparison of seven commercial SARS-CoV-2 rapid point-of-care antigen tests: a single-centre laboratory evaluation study

**DOI:** 10.1016/S2666-5247(21)00056-2

**Published:** 2021-07

**Authors:** Victor M Corman, Verena Claudia Haage, Tobias Bleicker, Marie Luisa Schmidt, Barbara Mühlemann, Marta Zuchowski, Wendy K Jo, Patricia Tscheak, Elisabeth Möncke-Buchner, Marcel A Müller, Andi Krumbholz, Jan Felix Drexler, Christian Drosten

**Affiliations:** a Institute of Virology, Charité-Universitätsmedizin Berlin, corporate member of Freie Universität Berlin, Humboldt-Universität zu Berlin, and Berlin Institute of Health, Berlin, Germany; b German Centre for Infection Research, Berlin, Germany; c Labor Berlin, Berlin, Germany; d Institute for Infection Medicine, Christian-Albrecht University and University Medical Center Schleswig-Holstein, Kiel, Germany; e Labor Dr Krause und Kollegen MVZ, Kiel, Germany

## Abstract

**Background:**

Antigen point-of-care tests (AgPOCTs) can accelerate SARS-CoV-2 testing. As some AgPOCTs have become available, interest is growing in their utility and performance. Here we aimed to compare the analytical sensitivity and specificity of seven commercially available AgPOCT devices.

**Methods:**

In a single-centre, laboratory evaluation study, we compared AgPOCT products from seven suppliers: the Abbott Panbio COVID-19 Ag Rapid Test, the RapiGEN BIOCREDIT COVID-19 Ag, the Healgen Coronavirus Ag Rapid Test Cassette (Swab), the Coris BioConcept COVID-19 Ag Respi-Strip, the R-Biopharm RIDA QUICK SARS-CoV-2 Antigen, the nal von minden NADAL COVID-19 Ag Test, and the Roche-SD Biosensor SARS-CoV Rapid Antigen Test. Tests were evaluated on recombinant SARS-CoV-2 nucleoprotein, cultured endemic and emerging coronaviruses, stored respiratory samples with known SARS-CoV-2 viral loads, stored samples from patients with respiratory pathogens other than SARS-CoV-2, and self-sampled swabs from healthy volunteers. We estimated analytical sensitivity in terms of approximate viral concentrations (quantified by real-time RT-PCR) that yielded positive AgPOCT results, and specificity in terms of propensity to generate false-positive results.

**Findings:**

In 138 clinical samples with quantified SARS-CoV-2 viral load, the 95% limit of detection (concentration at which 95% of test results were positive) in six of seven AgPOCT products ranged between 2·07 × 10^6^ and 2·86 × 10^7^ copies per swab, with an outlier (RapiGEN) at 1·57 × 10^10^ copies per swab. The assays showed no cross-reactivity towards cell culture or tissue culture supernatants containing any of the four endemic human coronaviruses (HCoV‑229E, HCoV‑NL63, HCoV‑OC43, or HCoV‑HKU1) or MERS-CoV, with the exception of the Healgen assay in one repeat test on HCoV-HKU1 supernatant. SARS-CoV was cross-detected by all assays. Cumulative specificities among stored clinical samples with non-SARS-CoV-2 infections (n=100) and self-samples from healthy volunteers (n=35; cumulative sample n=135) ranged between 98·5% (95% CI 94·2–99·7) and 100·0% (97·2–100·0) in five products, with two outliers at 94·8% (89·2–97·7; R-Biopharm) and 88·9% (82·1–93·4; Healgen). False-positive results did not appear to be associated with any specific respiratory pathogen.

**Interpretation:**

The sensitivity range of most AgPOCTs overlaps with SARS-CoV-2 viral loads typically observed in the first week of symptoms, which marks the infectious period in most patients. The AgPOCTs with limit of detections that approximate virus concentrations at which patients are infectious might enable shortcuts in decision making in various areas of health care and public health.

**Funding:**

EU's Horizon 2020 research and innovation programme, German Ministry of Research, German Federal Ministry for Economic Affairs and Energy, German Ministry of Health, and Bill & Melinda Gates Foundation.

## Introduction

The ongoing SARS-CoV-2 pandemic continues to challenge public health systems worldwide. In the absence of global vaccine availability or effective drugs, virus detection by real-time RT-PCR (RT-rtPCR) has been widely adopted to enable non-pharmaceutical interventions based on case finding and contact tracing. Because of its high sensitivity and specificity, RT-rtPCR is the gold standard for SARS-CoV-2 detection.^1^

RT-rtPCR is a laboratory-based procedure that requires sophisticated equipment, trained personnel, and logistical planning for sample shipment and results communication. Timeliness of results is crucial for the control of onward transmission, due to shedding of infectious virus mainly occurring around the time of symptom onset.^2^ However, delays in obtaining RT-rtPCR results are widespread, and they are compounded by the increasing demand for RT-rtPCR tests that are certified for in-vitro diagnostic application, which creates supply bottlenecks and a shortfall of overall testing capacity in many countries.^3^

Antigen detection tests for the identification of SARS-CoV-2 infection are considered inferior to RT-rtPCR in terms of sensitivity and specificity.^4^, ^5^ However, they offer the possibility of point-of-care testing, which can provide essential information when needed—an advantage that can offset the possibility, in some situations, of having to amend the information from an RT-rtPCR result obtained at a later point. As SARS-CoV-2 antigen point-of-care test (AgPOCT) devices are becoming available from various manufacturers, interest is growing in their performance, with particular regard to sensitivity and overall specificity, as two essential parameters that can guide decisions for application.^6^ Because of the intense but short-lived nature of SARS-CoV-2 shedding from the upper respiratory tract, the clinical validation of AgPOCTs requires a focus on the timing of infection in studied patients.^7^, ^8^ If patients are tested late in the course of infection, such as in the second week after symptom onset, incongruences between RT-rtPCR and AgPOCT will cause an apparently low clinical sensitivity of AgPOCTs that is not necessarily relevant to the use of these tests in diagnosing early acute infections.^9^ In addition to the fraction of virus-positive patients detected by the test (clinical sensitivity), sensitivity can also be expressed in terms of an antigen concentration limit, below which the capability of the test to detect an infected patient is lost (analytical sensitivity). From a practical perspective, knowledge of the analytical sensitivity rather than clinical sensitivity of AgPOCTs might be sufficient to judge their utility in various fields of application (eg, screening in outpatient departments and testing in the workplace or the general population), as compared with the well established reference method of RT-rtPCR.^10^

**Research in context**

**Evidence before this study**

We searched PubMed for studies published in English from database inception until Feb 1, 2021, on SARS-CoV-2 antigen point-of-care tests (AgPOCTs). We used the search terms “severe acute respiratory syndrome coronavirus 2”, “SARS-CoV-2”, “COVID-19”, “antigen”, “antigen test”, “point of care test”, and “lateral flow”. We found 37 studies that tested AgPOCTs. Most studies had measured the clinical performance of a single AgPOCT in a specific study population. A common limitation of many studies is insufficient diversity in AgPOCTs assessed in parallel. We identified only five studies evaluating more than two AgPOCTs, up to a maximum of four AgPOCTs.

**Added value of this study**

Our report provides a first comparative evaluation of SARS-CoV-2 rapid antigen tests available on the European market (per our criteria of availability in Europe in September, 2020). Our results include comparative estimates of limits of detection based on synthetic and natural viral proteins and prequantified patient samples. Specificity was assessed on the basis of clinical samples containing respiratory pathogens other than SARS-CoV-2 and fresh clinical samples from healthy patients.

**Implications of all the available evidence**

The overall findings enable a comparative assessment of sensitivity against real-time RT-PCR (RT-rtPCR), with a view to changes in practice in early case detection, and in decision criteria for the termination of isolation in infected patients. The limits of detection of most of the studied AgPOCTs approximate virus concentrations at which patients are infectious, justifying a reliable use in various areas of health care and public health, including decisions on immediate isolation measures. However, due to the lower sensitivity of AgPOCTs versus RT-rtPCR, they might not have the power to exclude SARS-CoV-2 infection in the very early and later phases of COVID-19.

In this study, we compared seven commercially available AgPOCT devices against an established RT-rtPCR assay,^11^ with the aim of estimating analytical sensitivity and specificity.

## Methods

### Study design and clinical samples

We did a single-centre evaluation in a laboratory setting. Evaluation of analytical sensitivity relied on recombinant SARS-CoV-2 nucleoprotein (SARS-CoV-2-N), SARS-CoV-2 cell culture supernatants, and anonymised stored clinical samples with established SARS-CoV viral loads. Specificity was evaluated on cell culture supernatants containing endemic and emerging human coronaviruses (HCoVs), anonymised stored clinical samples that had previously tested positive for respiratory pathogens other than SARS-CoV-2, and new naso-oropharyngeal self-swabs of healthy volunteers.

All stored clinical specimens were obtained during routine diagnostic testing with no extra procedures specific for the present study. Samples were chosen if of sufficient volume to be tested in at least three AgPOCTs and a SARS-CoV-2 RT-rtPCR assay. Anonymised nasopharyngeal or oropharyngeal swab specimens for specificity testing were obtained between Jan 2 and Dec 28, 2019, from multiple testing sites in Berlin, Germany (including all Charité-Universitätsmedizin Berlin centres and local communal hospitals), and sent to our diagnostic laboratories at Labor Berlin – Charité Vivantes and the Institute of Virology (Berlin, Germany) for routine diagnostic testing. Samples were tested with the NxTAG Respiratory Pathogen Panel (Luminex, Austin, TX, USA). Samples were chosen for the present study with a view to cover a broad diversity of respiratory pathogens typically detected in naso-oropharyngeal samples. Samples for specificity testing were stored in phosphate-buffered saline (PBS) or universal transport medium (UTM; Copan, Brescia, Italy) at −20°C. The SARS-CoV-2-positive samples for sensitivity testing were obtained between March and October, 2020, from multiple sites in Berlin and north Germany, and were sent to our laboratory at the Institute of Virology for SARS-CoV-2 testing or confirmation. These samples were received in UTM, PBS, or without any medium. Due to anonymisation, we had no other inclusion or exclusion criteria than SARS-CoV-2 RNA detection. All stored samples (including those from 2019) were retested and quantified for SARS-CoV-2 by RT-rtPCR as described previously.^11^, ^12^ Viral RNA of HCoVs other than SARS-CoV-2 was quantified by RT-rtPCR with specific in-vitro transcribed RNA standards.^11^, ^13^, ^14^, ^15^, ^16^ Details of the RNA extraction and RT-rtPCR testing procedures are given in the appendix (p 2).

The use of stored clinical samples for validation of diagnostic methods of anonymised data is covered by section 25 of the Berlin Hospital Law and does not require ethical or legal clearance. The ethical committee at Charité-Universitätsmedizin Berlin was notified of the study and acknowledged receipt under file number EA1/369/20.

### SARS-CoV-2-negative healthy volunteers

New self-swabs were obtained from volunteers with no respiratory symptoms who were employees of the Institute of Virology, between Oct 18 and Oct 30, 2021. We had no other eligibility criteria other than consent to be part of this study. All volunteers received instructions and materials to self-collect oropharyngeal or nasopharyngeal swabs (per test user instructions) and self-test with all AgPOCTs at a single timepoint (<1 h). All testing was done under supervision by medically trained personnel. Most manufacturers do not anticipate self-testing as per instructions for use. However, all volunteers were experienced laboratory personnel trained in liquid handling and other procedures similar to those required by AgPOCTs. Also, self-sampling was shown in recent months to be a reliable alternative to professional nasopharyngeal swabs for AgPOCT.^17^ All manufacturers' instructions were exactly followed during self-sampling. Volunteers used each test once. In addition to AgPOCT, one naso-oropharyngeal swab from each volunteer was tested by SARS-CoV-2 RT-rtPCR,^11^ with a negative result in all cases.

The testing of employees by AgPOCTs and RT-rtPCR is part of an ongoing study on SARS-CoV-2 infection in employees under Charité-Universitätsmedizin Berlin ethical review board file number EA1/068/20. All participants provided written informed consent.

### Recombinant SARS-CoV-2-N

The coding sequence of SARS-CoV-2-N was amplified, purified, and cloned into the expression vector pET151/D-TOPO (Thermo Fisher Scientific, Waltham, MA, USA) for expression of recombinant protein. *Escherichia coli* BL21 (DE3) cells were transformed with the pET151/D-TOPO–SARS-CoV-2 N plasmid. N protein was purified by affinity chromatography under native conditions as described previously.^18^ A second purification step was included with heparin sepharose columns (appendix p 3). Recombinant SARS-CoV-2 N was eluted with an NaCl gradient and protein concentration was determined photometrically. For analytical sensitivity experiments, N was diluted in PBS, obtaining dilutions between 2·5 ng/mL and 1000 ng/mL, and 50 μL of each dilution was applied to each AgPOCT. Three replicates per test were done.

### Cell culture samples

Pathogenic HCoVs, comprising all endemic HCoVs (ie, HCoV‑229E, HCoV‑NL63, HCoV‑OC43, and HCoV‑HKU1), MERS-CoV, SARS-CoV, and SARS-CoV-2, were grown in cell culture and the corresponding supernatants tested in duplicates.^19^, ^20^, ^21^, ^22^ RNA concentrations in all samples were determined by specific RT-rtPCR and in-vitro transcribed RNA standards designed for absolute quantification of viral load. Details of the cell culture and RT-rtPCR testing procedures are given in the appendix (pp 2, 4). In the case of SARS-CoV-2, additional quantification was done by plaque titration to obtain plaque-forming units for sensitivity testing.^12^

### AgPOCTs

We included seven AgPOCT products available to our laboratory at study initiation (late September, 2020): Panbio COVID-19 Ag Rapid Test (Abbott, Jena, Germany); BIOCREDIT COVID-19 Ag (RapiGEN, St Ingbert, Germany); Coronavirus Ag Rapid Test Cassette (Swab; Healgen, Houston, TX, USA); COVID-19 Ag Respi-Strip (Coris BioConcept, Gembloux, Belgium); RIDA QUICK SARS-CoV-2 Antigen (R-Biopharm, Darmstadt, Germany); NADAL COVID-19 Ag Test (nal von minden, Moers, Germany); and SARS-CoV Rapid Antigen Test (Roche-SD Biosensor, St Ingbert, Germany). We refer to the different test kits using the names of the manufacturers hereafter. During evaluation of the AgPOCTs, results in the form of a band on immunochromatography paper were scored independently by two authors (among VMC, TB, MLS, WKJ, and PT). In case of discrepant evaluations, a third person was consulted to reach a final decision (VMC, TB, or MLS). In case of test failure indicated by absence of a visible positive control band, the test procedure was repeated on the same sample.

Initial comparisons of analytical sensitivity relied on purified, bacterially expressed viral nucleocapsid protein (the target protein of all AgPOCTs). This testing was completed with diluted cell culture supernatants from SARS-CoV-2-infected Vero cells at defined concentrations of infectious (plaque-forming) units (PFUs) of virus (between around 0·044 PFUs and 440 PFUs per assay resulting from the serial dilutions; corresponding to dilutions between 0·88 PFU/mL and 8800 PFU/mL; table 1).

**Table 1:** Number of positive antigen point-of-care tests of serial dilutions of SARS-CoV-2 N and SARS-CoV-2 cell culture supernatant (triplicates)

	**Abbott**	**RapiGEN**	**Healgen**	**Coris BioConcept**	**R-Biopharm**	**nal von minden**	**Roche-SD Biosensor**
**SARS-CoV-2 N concentration, ng/mL**							
1000	3	3	3	3	3	3	3
250	3	3	3	3	3	3	3
50	3	0	3	3	3	3	3
25	3	0	3	3	3	3	3
10	3	0	3	0	3	3	3
5	2	0	3	0	3	2	3
2·5	0	0	3	0	3	0	0
**SARS-CoV-2, plaque-forming unit per mL**							
8800	3	3	3	3	3	3	3
880	3	2	3	3	3	3	3
88	3	0	3	0	3	1	3
8·8	0	0	0	0	0	0	0
0·88	0	0	0	0	0	0	0

Protein and virus were diluted in phosphate-buffered saline. 50 μL was used for testing. SARS-CoV-2 N=recombinant SARS-CoV-2 nucleoprotein.

To measure the analytical sensitivity in clinical samples with established SARS-CoV-2 viral loads, we used the stored swabs in UTM, PBS, or without any medium. Dry swabs were suspended in PBS. Of each suspension, 50 μL was introduced into the recommended volume of lysis reagent for each AgPOCT. Notably, this procedure with swabs diluted in buffer introduces a predilution step (approximately 1:20) not normally applied in AgPOCT protocols, resulting in a loss of sensitivity as opposed to RT-rtPCR. However, the swabs used for this study were standard-gauge flocked swabs that are not provided with the AgPOCTs. The swabs provided with the AgPOCTs consist of the same material but are considerably thinner and thus carry less sample volume. To estimate the relative sample input in the present procedure, we inserted standard flocked swabs and the swabs included in the AgPOCT kits in a solution of 50% sucrose and determined the relative sample volume contained in each swab by weighing the swabs. The resulting relative sample volume on AgPOCT swabs was around 40% (range 10–90%) of that in standard-gauge swabs. Taking our predilution step into account, this results in an approximately 8-fold lesser sample input in AgPOCT in the present study, as opposed to direct application as per manufacturer's instructions. This factor should be accounted for when directly comparing against the RT-rtPCR process and sensitivity herein. Also noteworthy is that the observable variability of swabs in some AgPOCT assays is considerable.

To identify any systematic cross-reactivity with relevant viral antigens, we tested cell cultures of HCoVs other than SARS-CoV-2, applying 50 μL of supernatant into the lysis buffer of each AgPOCT in triplicate. Furthermore, we used clinical samples negative for SARS-CoV-2 RNA but positive for other respiratory pathogens and samples taken from healthy volunteers with negative SARS-CoV-2 RT-rtPCR results for specificity testing. All negative samples that showed a SARS-CoV-2 false-positive result in AgPOCTs were retested and confirmed as false-positive with SARS-CoV-2 RT-rtPCR.

### Statistical analysis

All samples available for the various tests were used. A Bayesian binomial logistic regression analysis for assessment of analytical sensitivity in clinical samples was applied with the PyMC3 package in Python,^23^ to determine 50% and 95% limits of detection (with 95% highest posterior density intervals [95% HPDIs]), defined as the mean concentrations measured by RT-rtPCR at which 50% and 95% of results were positive, for each AgPOCT. The logistic regression model was implemented with the likelihood given as *y* ~ Bernoulli(θ), where y is the binary AgPOCT result (positive or negative); and the linear model was given as θ=logistic(α + β × *X*), where *X* is the observed log_10_ SARS-CoV-2 load measured as RNA copies per mL (equal to RNA copies per swab when resuspended in 1 mL fluid). The α variable refers to the intercept (determining the position of the inflection point on the x-axis) and the β variable to the slope (determining the steepness of the curve at the inflection point). For the distributions of α and β we used normal distributions with a mean of 0 and an SD of 15 (ie, α ~ normal[0, 15] and β ~ normal[0, 15]). Models were run for 25 000 generations with 5000 tuning steps, with the automatically assigned No-U-Turn sampler (Markov chain Monte Carlo method) and an acceptance rate of 0·95. Models were assessed for convergence via two criteria: the Gelman Rubin statistic, whereby all inferred parameters have values close to 1·0, and visualisations of posterior traces (example trace plots are given in the appendix, p 7). Posterior predictive distributions were used to assess model fit. An analysis of detection rate by the AgPOCTs was also done according to SARS-CoV-2 concentrations classified into three viral load categories (appendix p 5).

Cumulative specificity was estimated from the exclusivity testing of stored samples from patients acutely infected with known respiratory pathogens other than SARS-CoV-2, and the testing of self-sampled swabs from healthy volunteers. The overall proportion of negative tests for each AgPOCT were calculated, with use of the first test result for false-positive tests that were repeated. 95% CIs for proportions were calculated by the Wilson procedure with a correction for continuity.^24^

### Role of the funding source

The funders of the study had no role in study design, data collection, data analysis, data interpretation, or writing of the report.

## Results

On testing of recombinant SARS-CoV-2 N dilutions, protein concentrations between 5 ng/mL and 25 ng/mL were detectable by most AgPOCT assays, corresponding to 250–1250 pg protein per 50 μL sample volume (table 1). We also tested the AcPOCTs with cell culture supernatants from SARS-CoV-2-infected Vero cells at defined PFUs of virus. Almost all AgPOCTs reliably detected around 44 PFU of virus per assay, corresponding to 880 PFU/mL (table 1). The assays by manufacturers Abbott, Healgen, R-Biopharm, and Roche-SD Biosensor reliably detected as little as 4·4 PFU of virus per test (88 PFU/mL). The RapiGEN assay was considerably less sensitive than the other assays in detecting recombinant protein or virus.

**Figure 1:**
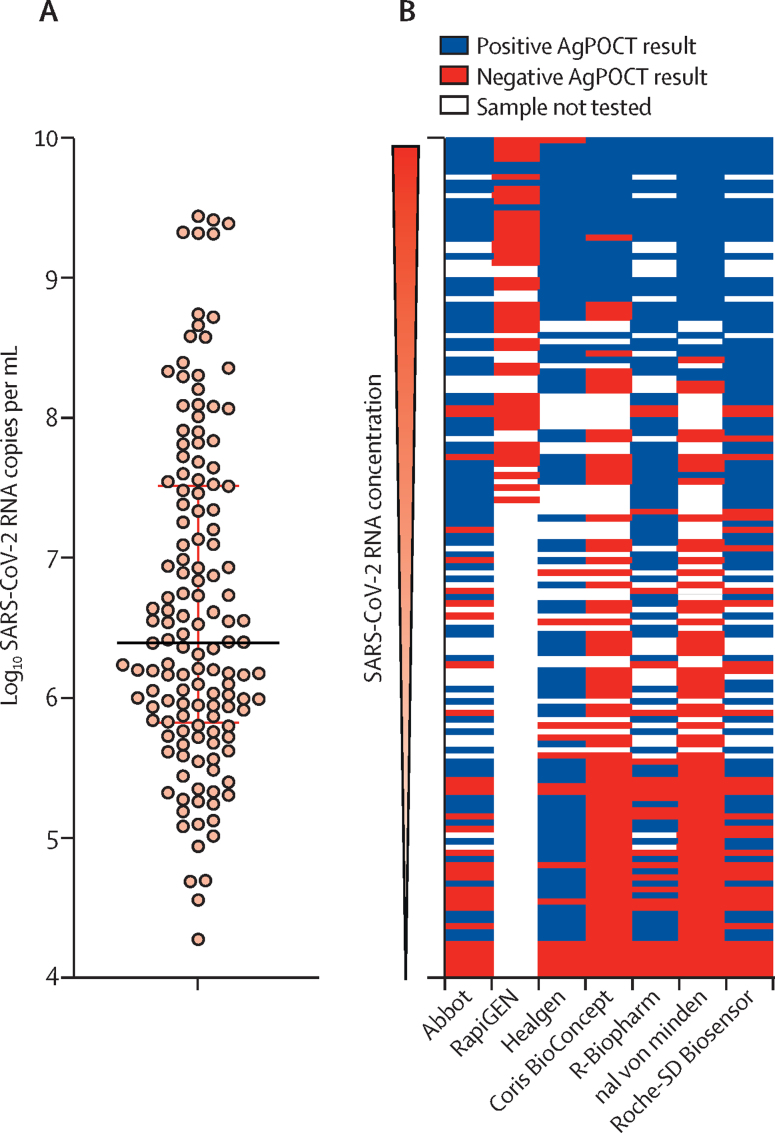
SARS-CoV-2 viral RNA concentrations in clinical samples (n=138) (A) Distribution of SARS-CoV-2 viral RNA concentrations after AgPOCT testing. Horizontal lines represent the median and IQR. (B) Overview of tested samples and corresponding outcomes in the seven AgPOCTs. AgPOCT=antigen point-of-care test.

We tested 138 clinical samples that had previously tested positive for SARS-CoV-2 by RT-rtPCR (figure 1A). Median virus load was 2·49 × 10^6^ RNA copies per mL (range 1·88 × 10^4^–2·75 × 10^9^) of swab suspension (figure 1A). Depending on available volume per clinical sample, up to 115 clinical samples per assay were used to evaluate AgPOCT assays. Only 45 samples were used for the RapiGEN assay, which detected only four of 45 samples correctly, with each of these four samples containing more than 2 × 10^8^ RNA copies per mL, leading us to terminate testing of more clinical samples for this product. The distribution of test samples and outcomes across all AgPOCT products is shown in figure 1B.

**Figure 2:**
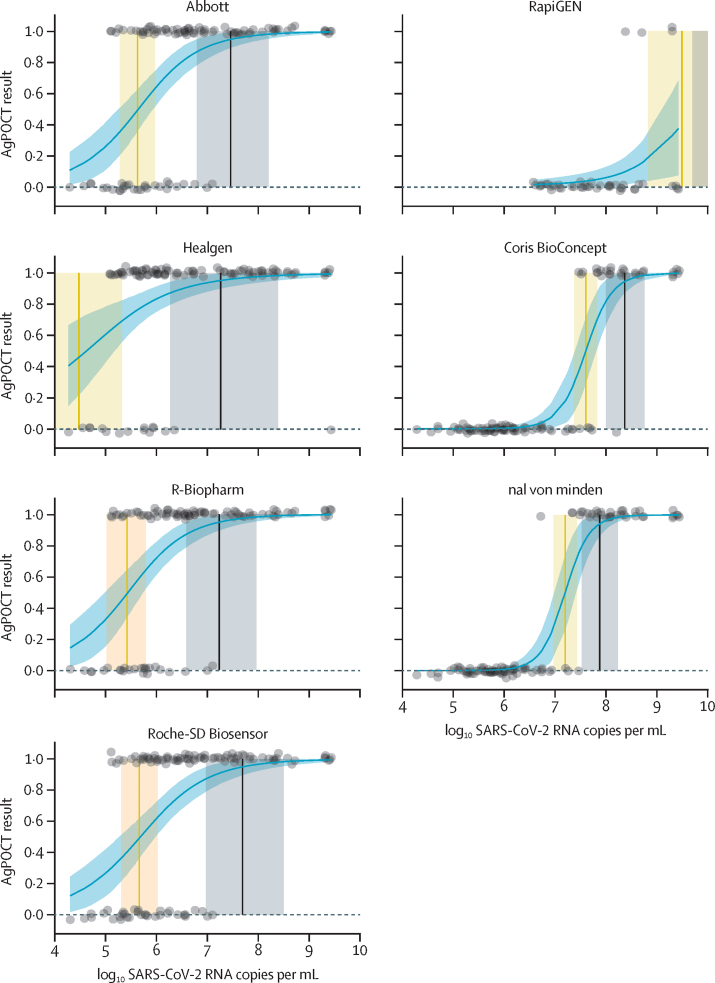
Predicted AgPOCT results as a function of log_10_ SARS-CoV-2 RNA per mL Datapoints show log_10_ SARS-CoV-2 RNA per mL (with jitter added on the y-axis) against positive (1·0) and negative (0·0) AgPOCT results. The dashed line and accompanying shaded region represent the mean and 95% HPDI of the Bayesian binomial logistic regression curve fitted to the data. The vertical lines (shaded areas) correspond to the mean (95% HPDI) SARS-CoV-2 concentrations (log_10_ RNA copies per mL) at which 50% (orange) and 95% (black) of samples have a positive AgPOCT result (1·0; table 1). Model fit for the RapiGEN and Healgen tests was poor, due to a large difference in the number of positive and negative test results. AgPOCT=antigen point-of-care test. HPDI=highest posterior density interval.

Without correction for the lower sample input in our study, as opposed to standard AgPOCT protocols, the virus concentrations quantified by RT-rtPCR at which 95% detection rates were achieved with AgPOCTs ranged between 1·66 × 10^7^ SARS-CoV-2 RNA copies per swab (95% HPDI 3·72 × 10^6^–9·12 × 10^7^) and 2·29 × 10^8^ SARS-CoV-2 RNA copies per swab (1·00 × 10^8^–5·75 × 10^8^; corresponding to 7·22 log_10_ SARS-CoV-2 RNA copies per swab [95% HPDI 6·57–7·96] and 8·36 log_10_ SARS-CoV-2 RNA copies per swab [8·00–8·76]; table 2) for the six most sensitive assays (Abbott, Healgen, R-Biopharm, nal von minden, and Roche-SD Biosensor; figure 2, appendix p 5). With correction for sample input, these numbers were lowered 8-fold, ranging between 2·07 × 10^6^ copies per swab and 2·86 × 10^7^ copies per swab. As expected, the RapiGEN test was an outlier, at 1·57 × 10^10^ copies per swab (table 2). The non-adjusted virus concentrations at which 50% detection rates were achieved ranged between 4·48 log_10_ SARS-CoV-2 RNA copies per swab [3·41–5·32] and 7·60 log_10_ SARS-CoV-2 RNA copies per swab [7·37–7·82] for the six most sensitive assays. The RapiGEN test (50% limit of detection at 9·51 log_10_ SARS-CoV-2 RNA copies per swab [8·84–12·26]; table 2) showed no positive results for 24 samples with RNA concentrations lower than 8·00 log_10_ SARS-CoV-2 RNA copies per swab (appendix p 5).

**Table 2:** Limits of detection

	**Number of tested samples**	50% limit of detection*(log_10_ SARS-CoV-2 RNA copies per swab†**)**	95% limit of detection*(log_10_ SARS-CoV-2 RNA copies per swab†**)**	Adjusted and converted 95% limit of detection*‡**(SARS-CoV-2 RNA copies per swab**§**)**
Abbott	105	5·61 (5·27–5·95)	7·45 (6·79–8·20)	3·52 × 10^6^
RapiGEN¶	45	9·51 (8·84–12·26)	11·10 (9·71–17·01)	1·57 × 10^10^
Healgen¶	105	4·48 (3·41–5·32)	7·27 (6·27–8·40)	2·33 × 10^6^
Coris BioConcept	105	7·60 (7·37–7·82)	8·36 (8·00–8·76)	2·86 × 10^7^
R-Biopharm	105	5·40 (4·99–5·77)	7·22 (6·57–7·96)	2·07 × 10^6^
nal von minden	105	7·19 (6·97–7·43)	7·87 (7·52–8·23)	9·27 × 10^6^
Roche-SD Biosensor	115	5·64 (5·28–6·00)	7·68 (6·96–8·50)	5·98 × 10^6^

AgPOCT=antigen point-of-care test.

* Mean concentration that yields 50% or 95% positive AgPOCT results according to a binary logistic regression analysis; numbers in parentheses denote the 95% highest posterior density interval determined by the Bayesian binary logistic regression model.

† Concentration per swab presumes that swabs are resuspended in 1 mL fluid during pre-analytical processes in RT-PCR used to measure viral loads.

‡ Due to a systematic pre-analytical dilution factor in our AgPOCT evaluations, the projected mean concentrations at which 95% hit rates were achieved were corrected to be 8-fold lower (a cumulative correction factor representing all correction factors between the actual volume input in our validation studies and the volume input as per manufacturer's instructions); input volumes in all cases are subject to great variability due to the undefined volumes of viscous respiratory tract specimens taken up by swab sampling devices, and our statistical evaluation suggests a level of precision that does not reflect the clinical reality in AgPOCT use.

§ Values have been converted to non-logarithmic number, as normally reported in clinical practice.

¶ Model fit was suboptimal due to a large difference in the number of positive and negative test results (figure 2).

None of the assays showed cross-reactivity towards cell culture supernatants containing any of the four endemic human coronaviruses (HCoV‑229E, HCoV‑NL63, HCoV‑OC43, and HCoV‑HKU1) or MERS-CoV, with the exception of the Healgen in one repeat test on HCoV-HKU1 supernatant. SARS-CoV was cross-detected by all assays (appendix p 5).

We tested 100 stored clinical samples from patients with known acute infections caused by respiratory pathogens other than SARS-CoV-2, including some samples containing mycoplasma and legionella. All assays detected either none, one, or two SARS-CoV-2 false-positive results in 100 samples, with the exception of Healgen, which detected 12 false-positive results (table 3). About half of all false-positive results were reproducible on retesting of the same sample, although we observed no obvious association between false-positive results and any specific known pathogen in the samples. Thus, a specific factor other than the tested pathogens was likely to have caused positive signals. In a total of 15 samples that tested false positive, one sample caused a positive signal in two different assays.

**Table 3:** Specificity in testing of clinical samples without SARS-CoV-2

		**Number of samples**	**Abbott**	**RapiGEN**	**Healgen**	**Coris BioConcept**	**R-Biopharm**	**nal von minden**	**Roche-SD Biosensor**
Pathogen									
	Adenovirus	9	0	0	1*	0	0	0	0
	Bocavirus	9	0	0	0	0	0	0	0
	HCoV-NL63	1	0	0	0	0	0	0	0
	HCoV-OC43	1	0	0	0	0	0	0	0
	Enterovirus or rhinovirus	9	0	0	1*	0	0	0	0
	Influenza virus A H1	10	0	0	2*	0	1†	0	0
	Influenza virus A H3	9	0	0	2‡	0	1†	0	0
	Influenza virus B	1	0	0	0	0	0	0	0
	Metapneumovirus	1	0	0	0	0	0	0	0
	HPIV-1	8	0	0	3*	0	0	0	0
	HPIV-2	3	0	0	2‡	0	0	0	0
	HPIV-3	10	0	0	1*§	0	0	0	1†§
	RSV-A	7	1*	0	0	0	0	0	0
	RSV-B	7	0	0	0	0	0	0	0
	Mycoplasma pneumoniae	8	0	0	0	0	0	0	0
	Legionella pneumophila	7	0	0	0	0	0	0	0
Total		100	1	0	12	0	2	0	1

Data indicate the number of SARS-CoV-2 false-positive results for each antigen point-of-care test. HCoV=human coronavirus. HPIV=human parainfluenza virus. RSV=respiratory syncytial virus.

* Non-specific positive reaction was reproduced in a repeat test for the false-positive sample or samples.

† Non-specific positive reaction was not reproduced in a repeat test.

‡ Non-specific positive reaction was reproduced in a repeat test for one of the two false-positive samples (reaction not reproduced for the other false-positive sample).

§ The same sample tested positive in the Healgen and Roche-SD Biosensor assays.

We further did a self-testing exercise with all of the AgPOCTs, enrolling 35 healthy laboratory employees, aged between 22 and 61 years (median 33 years [IQR 28–39]), without symptoms of respiratory tract infection and who tested negative for SARS-CoV-2 RNA by RT-rtPCR. The same AgPOCTs that generated false-positive results with stored clinical samples also showed positive signals in healthy volunteers, except for the Abbott test, which detected no positive signals in healthy volunteers. Conversely, the nal von minden test, showing no false positives in clinical samples, detected a positive signal in one volunteer sample (appendix p 6). All positive results were resolved to false-positive with parallel testing by RT-rtPCR.

The cumulative specificities from exclusivity testing and testing of healthy volunteers (cumulative sample n=135) were: for the Abbott test, 99·3% (95% CI 95·3–100·0; 134 true negatives); for the RapiGEN test, 100·0% (97·2–100·0; 135 true negatives); for the Healgen test, 88·9% (82·1–93·4; 120 true negatives); for the Coris BioConcept test, 100·0% (97·2–100·0; 135 true negatives); for the R-Biopharm test, 94·8% (89·2–97·7; 128 true negatives); for the nal von minden test, 99·3% (95·3–100·0; 134 true negatives); and for the Roche-SD Biosensor test, 98·5% (94·2–99·7; 133 true negatives). Given these values, the R-Biopharm and Healgen tests were considered as outliers.

## Discussion

We provide a comparison of performance of seven AgPOCT assays that have become available on the European market in recent months. These medical diagnostic devices are cleared in many countries for use outside the laboratory, provided that testing results are supervised by medical personnel. The short turnaround time of these tests is expected to enable major changes in clinical and public health practice, assuming that sensitivity and specificity is sufficient. Because of the strong demand during a constantly evolving situation, the question of sensitivity and specificity has not been thoroughly clarified for most AgPOCT products.

The aim of the present study was to ease some of the challenges associated with the clinical evaluation of AgPOCTs during the current pandemic situation. As the arrival of prototype tests coincided with a time of low COVID-19 incidence during the summer months in the northern hemisphere, the recruitment of patients newly infected with SARS-CoV-2 for clinical evaluation was difficult. Due to the rapid change of viral load in the acute phase of COVID-19,^12^, ^25^ AgPOCTs have a narrow timeframe for their useful application that predominantly comprises the first week of symptoms. In view of the increasing experience with RT-rtPCR testing during this timeframe, we aimed to mainly provide a reflection of AgPOCT performance on the basis of analytical properties; specifically, the approximate viral concentrations that can be detected by the assays and their propensity to generate false-positive results.

In terms of analytical sensitivity, the detection range of most AgPOCTs was found to range between around 2 million and 9 million copies per swab (accounting for a systematic predilution), and thus corresponds to a concentration that can be expected to yield a virus isolation success rate of around 20% in cell culture.^12^, ^26^, ^27^, ^28^ Based on previous studies,^12^, ^26^, ^27^, ^28^ this rate of isolation success would typically be reached by the end of the first week of symptoms. He and colleagues^25^ have shown that this point in time also correlates with the end of the period during which infected individuals can transmit the virus. Although many caveats remain, the point in the course of the first week of symptoms at which AgPOCT results turn negative might thus indicate the time at which infectivity resolves.^29^ The immediate availability of test results could enable novel public health concepts in which decisions to isolate or maintain isolation are based on infectivity testing rather than infection screening. On first patient contact, a positive AgPOCT result could also help to decide on immediate isolation measures by the identification of individuals who shed particularly large amounts of virus, which would be particularly useful in emergency departments.^30^ In hospitalised patients at the end of their clinical course, AgPOCT results might provide an additional criterion to maintain or modify isolation precautions.

Screening of asymptomatic patients with the expectation of being able to discern between virus absence and asymptomatic or presymptomatic infection is more difficult. Given the limitations in sensitivity, the results of AgPOCTs should be understood as a momentary assessment of infectiousness, instead of a diagnosis with power to exclude infection. As a steep increase in virus concentration occurs around or before the onset of symptoms, guidelines for AgPOCTs should mention that a negative test result might reflect low sensitivity, particularly as symptoms could occur soon after testing. Instructions that limit the validity of a negative test result in healthy patients to the day of application could be used to address this challenge.

The limited specificity of some AgPOCTs might also need to be amended by RT-rtPCR confirmation if resources permit. We observed acceptable rates of false-positive results (<3%) with most AgPOCTs, but rates greater than 5% with two assays in particular. One of these assays (R-Biopharm) was tested as a preliminary version predating the marketed product. The other assay (Healgen) might be limited by lot-to-lot variability, as an independent study of the same product did not show similar issues with false-positive results (as reported in the product insert of the device).

Our study has some limitations. We can only provide an approximate sensitivity assessment for individual AgPOCTs as we used stored samples and had to apply equal pre-analytical treatments despite slight differences between kits in terms of the size of the swab samples. An absolute assessment of limits of detection for each test, and a strict comparison of relative sensitivities, are therefore not possible. Additionally, the encountered issues with specificity of two products are unlikely to persist and might be explained by the testing of early production lots in this study. However, false-positives do occur with AgPOCTs at a higher rate than with RT-rtPCR. Our study also does not compare practical differences between assays, for instance, whether sample buffers are provided as a bulk volume or are prefilled in reaction tubes. These issues will need to be addressed for the qualification of products as consumer-grade (home) tests, a process that is underway for some but not all products. There are other limitations, including the absence of clinical information due to anonymisation of samples.

Overall, the present contribution provides an early impression of the performance of AgPOCTs from several major distributors. The sensitivity range of most investigated AgPOCTs (except for RapiGEN) overlaps with viral load figures from the infectious period in most patients. Consequently, most of these assays could potentially be used in efforts to limit transmission, but due to their lower sensitivity than RT-rtPCR, AgPOCTs might not have the power to exclude SARS-CoV-2 infection in the very early and later phases of COVID-19. Clinical validation and studies are necessary to confirm the observed sensitivity and specificity, and to incorporate them into clinical guidelines.

## Data sharing

Raw viral load data for tested samples can be obtained from the corresponding author on request.

## Declaration of interests

We declare no competing interests.
