# Supplementary material for: Comparison of seven commercial SARS-CoV-2 rapid point-of-care antigen tests: a single-centre laboratory evaluation study
Source: Lancet Microbe. 2021 Jul;2(7):e311–9. doi: 10.1016/S2666-5247(21)00056-2 (PMC8026170; doi:10.1016/S2666-5247(21)00056-2)
Supplement: Supplementary appendix [file mmc1.pdf]

THE LANCET
Microbe

Supplementary appendix

This appendix formed part of the original submission and has been peer reviewed. We post it as supplied by the authors.

Supplement to: Corman V M, Haage V C, Bleicker T, et al. Comparison of seven commercial SARS-CoV-2 rapid point-of-care antigen tests: a single-centre laboratory evaluation study. *Lancet Microbe* 2021; published online April 7. https://doi.org/10.1016/S2666-5247(21)00056-2.

Table of contents

1. **Supplementary materials and methods**
2. **Supplementary Table S1: Hit rates by viral load in test samples**
3. **Supplementary Table S2. Specificity in testing using cell culture supernatants of other human coronaviruses.**
4. **Supplementary Table S3: False positive results in 35 SARS-CoV-2 negative employees**
5. **Supplementary Figure 1: Example trace plots of parameters alpha and beta, and the 50% (bd05) and 95% (bd95) limits of detection**

1. Supplementary materials and methods

RT-PCR testing

Diagnostic SARS-CoV-2 RT-PCR testing was performed with the Cobas SARS-CoV-2 assay (Roche, Penzberg, Germany) on the Roche cobas 6800 or 8800 system or the Roche MagNA Pure 96 System for RNA purification and the SARS-CoV-2 E-gene assay from TibMolbiol (Berlin, Germany). All samples were retested and re-quantified by RT-PCR targeting the E-Gen of SARS-CoV-2 specifically for this study. RNA was extracted from clinical samples by using the MagNA Pure 96 system (Roche, Penzberg, Germany). The viral RNA extraction was performed using 100µl of sample, eluted in 100µl.

Detection of four endemic Coronaviruses namely HCoV-229E, HCoV-OC43, HCoV-NL63, HCoV-HKU1 a 25 µl reaction was set up containing 5 µl of RNA, and the QIAGEN OneStep RT-PCR Kit(Qiagen, Hilden, Germany).The amplification procedure comprised reverse transcription at 55°C for 20 min followed by initial denaturation at 94°C for 3 min and 45 cycles of 94°C for 15 s and 58°C for 30 s. Detection of MERS-CoV, SARS-CoV, and SARS-CoV-2 was done as described elsewhere using the upE for MERS-CoV and the E-gene target for SARS-CoV and SARS-CoV-2, respectively[1,2].Assessment of viral RNA concentration was done by applying internal calibration curves and using serial diluted assay-specific photometrically quantified in-vitro transcribed RNA[1-3].

The sequences of primers and probes for each assay are described elsewhere [4] [5]. All RT-PCR's were performed using a LightCycler 480 II (Roche, Germany).

Recombinant SARS-CoV-2 nucleoprotein (SARS-CoV-2-N)

The coding sequence of the SARS-CoV-2 nucleoprotein was amplified, purified and cloned into the expression vector pET151/D-TOPO (Thermo Fisher Scientific). E. coli BL21 Star™(DE3) One Shot® cells (Thermo Fisher Scientific, catalog no. C6010-03) were transformed with the pET151/D-TOPO-SARS-CoV-2 N plasmid. Protein purification was performed by Ni-NTA affinity chromatography under native conditions as described previously with modifications[6]. Briefly, E. coli cells (500 ml volume) were grown in LB medium to an optical density of 0.6, induced with 0.5 mM isopropyl β-D-1-thiogalactopyranoside (IPTG) and incubated at 37°C for 4 hours. After pelleting, cells were lysed for 1 hour at 4°C in lysis buffer (50 mM NaH2PO4 pH 8.0; 300 mM NaCl) supplemented with 10 mM imidazole, 1 x BugBuster Protein Extract Reagent (Millipore), 1 x EDTA-free protease inhibitor cocktail cOmplete tablets (Roche), 25 units/ml benzonase (Millipore) and 0,5 mg/ml lysozyme (Serva). The lysate was centrifuged at 4°C and 20.000 x g for 40 minutes and the supernatant fraction was mixed with 2 ml Ni-NTA resin (Qiagen) which was pre-washed twice with 10 volumes lysis buffer, gently rotated at 4°C for 2 hours and then poured to a polypropylene column (Qiagen). After washing four times with lysis buffer supplemented with 10 mM and 50 mM imidazole, respectively, proteins were eluted using lysis buffer supplemented with 500 mM imidazole. For a second purification step, fractions with the highest amount of SARS-CoV-2 N protein were pooled and loaded onto a heparin sepharose column (1 ml HiTrapHeparin, GE Healthcare) which was equilibrated with 5 column volumes (CV) of buffer A (20 mM Tris-HCl pH 7.5, 0.1 mM EDTA, 100 mM NaCl buffer). After washing with 5 CV of buffer A, the N protein was eluted by 10 CV of a 100-1000 mM NaCl linear gradient in buffer A in 500 µl fractions at a flow rate of 0,5 ml/min. The eluted protein fractions were subsequently dialysed twice against 500-fold volume of PBS for >8 h (molecular weight cut off: 10 kDa). For analytical sensitivity, experiments SARS-CoV-2-N protein was diluted in PBS and 50 µl of each dilution were applied to each test. Three replicates per test were performed.

Cell culture samples

Human pathogenic CoV (HCoV) were grown on cell monolayers at 37°C using DMEM/10%FBS at 5% CO2 for 4-7 days as described previously[7-10]. In detail, HCoV-229E (Inf-1) and OC43 (VR-759) were grown on Huh-7D12 cells (ECACC 01042712; RRID:CVCL_2957) for 4 and 7 days, respectively, as described before[8], -NL63 (Amsterdam) on Caco-2 (ATCC HTB-37; RRID:CVCL_0025) for 4 days[10], -HKU1 (Caen-1) on primary human air liquid interface cell cultures for 5 days as described previously[11]. MERS-CoV (EMC) was grown on VeroB4 (DZMZ ACC-33; RRID:CVCL_1912)[7], SARS-CoV (Frankfurt-1)[9] as well as SARS-CoV-2 (strain BetaCoV/Munich/ChVir984/2020)[12] on VeroE6 (ATCC:CRL-1586; RRID:CVCL_0574) cells for 4 days. For quantification, viral RNAs were extracted from cell culture supernatants by the viral RNA mini kit (Qiagen) according to the manufacturer's instructions at the above-mentioned time points. RNA concentrations in all samples were determined by specific real-time RT-PCR and in vitro-transcribed RNA standards as described above. In case of SARS-CoV-2, additional quantification was done by plaque titration[12]. In case of MERS-CoV, SARS-CoV and SARS-CoV-2 virus stock productions as well as applications of native supernatants were done under BSL3 conditions wearing full protective safety equipment. All cell culture supernatants containing HCoV-229E, -NL63, -OC43 and -HKU1 as well as MERS-CoV, SARS-CoV, and SARS-CoV-2 were tested in duplicates.

Table S1. Hit rates by viral load in test samples

Range of RNA Concentration [RNA copies/mL]	$N_{positive}/N_{tested}$/ (%; 95%CI)						
	Abbott	RapiGEN	Healgen	Coris	R-Biopharm	Nal von minden	Roche/SD Biosensor
10^8 - 10^10	16/16 (100.00; 75.93-100)	4/21 (19.05; 6.29-42.58)	21/22 (95.45; 75.11-99.76)	21/22 (95.45; 75.11-99.76)	16/16 (100.00; 75.93-100.00)	22/22 (100.00; 81.5-100.00)	16/16 (100.00; 75.93-100.00)
10^6 - <10^8	41/50 (82.00; 68.08-90.95)	0/24 (0.00; 0.00-17.17)	38/40 (95.00; 81.79-99.13)	8/40 (20.00; 9.61-36.14)	45/50 (90.00; 77.41-96.26)	14/40 (35.00; 21.10-51.74)	46/58 (79.31; 66.28-88.41)
10^4 - <10^6	18/39 (46.15; 30.43-62.62)	-	30/43 (69.77; 53.70-83.33)	0/43 (0.00; 0.00-10.21)	19/39 (48.72; 32.71-64.97)	0/43 (0.00; 0.00-10.21)	19/41 (46.34; 30.97-62.39)

Table S2. Specificity in testing using cell culture supernatants of other human coronaviruses.

		AgPOCT assay[a]						
Virus	Concentration [RNA copies/mL]	Abbott	RapiGEN	Healgen	Coris	R-Biopharm	Nal von minden	Roche/SD Biosensor
HCoV-229E	2.87E+07	0/2	0/2	0/2	0/2	0/2	0/2	0/2
HCoV-OC43	1.0E+06	0/2	0/2	0/2	0/2	0/2	0/2	0/2
HCoV-NL63	1.70E+06	0/2	0/2	0/2	0/2	0/2	0/2	0/2
HCoV-HKU1	1.30E+07	0/2	0/2	1/3	0/2	0/2	0/2	0/2
MERS-CoV	1.87E+08	0/2	0/2	0/2	0/2	0/2	0/2	0/2
SARS-CoV	2.12E+09	2/2	2/2	2/2	2/2	2/2	2/2	2/2

[a]Tests were performed by using non-inactivated cell culture supernatants in duplicates.

Table S3. False positive results in 35 SARS-CoV-2 negative employees

AgPOCT[a]	Abbott	RapiGEN	Healgen	Coris	R-Biopharm	Nal von minden	Roche/SD Biosensor
False positives	-	-	3[b]	-	5[b]	1	1
Specificity (%)[b,c]	100 (95%CI: 87.68-100.00)	100 (95%CI: 87.68-100.00)	91.43 (95%CI: 75.82-97.76)	100 (95%CI: 87.68-100.00)	85.71 (95%CI: 68.95-94.62)	97.14 (95%CI: 83.38-99.85)	97.14 (95%CI: 83.38-99.85)

[a]In 35 subjects, 30 conducting nasopharyngeal swabs and 5 conducting pharyngeal swabs

[b]One person tested false positive in assays Healgen and R-Biopharm.

[c] CI; 95% confidence interval were calculated using the Wilson procedure with a correction for continuity[13].

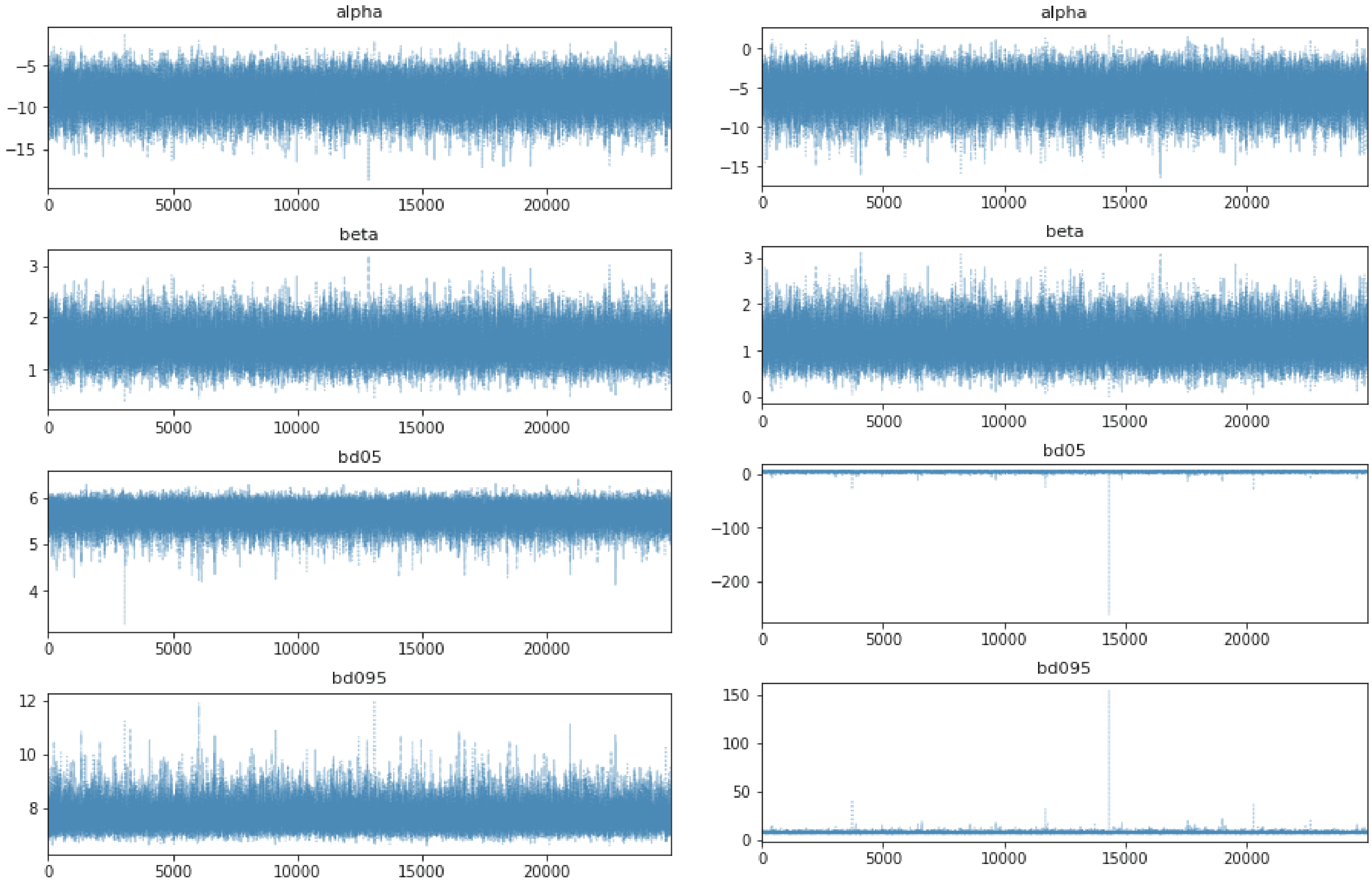

Supplementary Figure 1: Example trace plots of parameters alpha and beta, and the 50% (bd05) and 95% (bd95) limits of detection, respectively, from top to bottom. The x-axis indicates the generation, the y-axis the parameter value. Left panel: Trace plot for test Roche/SD Biosensor, as an example of convergence. Right panel: Trace plot for test Healgen, where convergence is not satisfactory for bd05 and bd95. As indicated in Table 2, model fit for this test was suboptimal due to a large difference in the number of positive and negative test results.

Supplementary References:

1. Corman VM, Eckerle I, Bleicker T, et al. Detection of a novel human coronavirus by real-time reverse-transcription polymerase chain reaction. *Euro Surveill* 2012; **17**(39).
2. Corman VM, Landt O, Kaiser M, et al. Detection of 2019 novel coronavirus (2019-nCoV) by real-time RT-PCR. *Euro Surveill* 2020; **25**(3).
3. Owusu M, Annan A, Corman VM, et al. Human coronaviruses associated with upper respiratory tract infections in three rural areas of Ghana. *PLoS One* 2014; **9**(7): e99782.
4. Dare RK, Fry AM, Chittaganpitch M, Sawanpanyalert P, Olsen SJ, Erdman DD. Human coronavirus infections in rural Thailand: a comprehensive study using real-time reverse-transcription polymerase chain reaction assays. *J Infect Dis* 2007; **196**(9): 1321-8.
5. van Elden LJ, van Loon AM, van Alphen F, et al. Frequent detection of human coronaviruses in clinical specimens from patients with respiratory tract infection by use of a novel real-time reverse-transcriptase polymerase chain reaction. *J Infect Dis* 2004; **189**(4): 652-7.
6. Dijkman R, Jebbink MF, El Idrissi NB, et al. Human coronavirus NL63 and 229E seroconversion in children. *J Clin Microbiol* 2008; **46**(7): 2368-73.
7. Muller MA, Raj VS, Muth D, et al. Human coronavirus EMC does not require the SARS-coronavirus receptor and maintains broad replicative capability in mammalian cell lines. *mBio* 2012; **3**(6).
8. Corman VM, Eckerle I, Memish ZA, et al. Link of a ubiquitous human coronavirus to dromedary camels. *Proc Natl Acad Sci U S A* 2016; **113**(35): 9864-9.
9. Niemeyer D, Mosbauer K, Klein EM, et al. The papain-like protease determines a virulence trait that varies among members of the SARS-coronavirus species. *PLoS Pathog* 2018; **14**(9): e1007296.
10. Herzog P, Drosten C, Muller MA. Plaque assay for human coronavirus NL63 using human colon carcinoma cells. *Virol J* 2008; **5**: 138.
11. Jonsdottir HR, Marti S, Geerts D, Rodriguez R, Thiel V, Dijkman R. Establishment of Primary Transgenic Human Airway Epithelial Cell Cultures to Study Respiratory Virus-Host Interactions. *Viruses* 2019; **11**(8).
12. Wolfel R, Corman VM, Guggemos W, et al. Virological assessment of hospitalized patients with COVID-2019. *Nature* 2020; **581**(7809): 465-9.
13. Newcombe RG. Two-sided confidence intervals for the single proportion: comparison of seven methods. *Stat Med* 1998; **17**(8): 857-72.
